# Herbal does not mean harmless: A case of turmeric-associated AGEP

**DOI:** 10.1016/j.jdcr.2026.08.017

**Published:** 2026-08-18

**Authors:** Madeleine Dumke, Zaina Rashid, Vian Al-Rubaie, Hongyu Yang

**Affiliations:** a Midwestern University Arizona College of Osteopathic Medicine, Glendale, Arizona; b La Peau Dermatology, Mesa, Arizona; c Tri-State Pathology Associates, St Vincent Evansville Hospital, Evansville, Indiana

**Keywords:** acute generalized exanthematous pustulosis, cutaneous adverse drug reaction, dietary supplements, drug-induced eruption, turmeric

## Introduction

Turmeric (*Curcuma longa*) is a widely used spice and dietary supplement promoted for its anti-inflammatory properties.^1^^,^^2^ Its popularity has grown substantially with increasing interest in natural health remedies.^3^^,^^4^ Despite widespread use, regulation of turmeric supplements remains limited, and commercial preparations vary considerably in curcuminoid content and labeling accuracy.^5^

Although rare, cutaneous hypersensitivity reactions to turmeric have been reported, including 2 prior cases of acute generalized exanthematous pustulosis (AGEP) and 1 vesicular drug eruption.^6^, ^7^, ^8^ Including the present report, only 4 turmeric-associated hypersensitivity reactions have been reported (Table I).

**Table I:** Published cases of turmeric-associated AGEP and cutaneous hypersensitivity reactions

Author/year	Age/sex	Turmeric dose	Time to onset	Clinical presentation	Histology	EuroSCAR	Treatment/outcome	Confirmatory testing
Wu et al 2020	81/F	1200 mg/d	1 mo	Diffuse nonfollicular pustules with fever	Neutrophilic spongiform pustules with mixed dermal inflammatory infiltrate	8 (Definite AGEP)	Resolved after discontinuation and topical steroids	Positive lymphocyte transformation test
Riva et al 2024	55/F	Increased from 500 to 1500 mg/d	2 d	Erythematous plaques with peripheral pustules	Subcorneal pustules with eosinophils	8 (Definite AGEP)	Resolved after discontinuation, oral and topical steroids	Not performed
Ito et al 2006	57/M	Not specified	2 y	Generalized pruritic erythema with vesicles	Spongiosis with dermal eosinophils	Not reported	Resolved after discontinuation and oral steroids	Positive patch test
Present case	86/F	4500 mg/d	3 d	Erythema with scattered resolving monomorphic pustules	Subcorneal pustular dermatitis with neutrophils	8 (Definite AGEP)	Resolved after discontinuation, intramuscular and topical steroids, and antihistamines	Allergy evaluation with patch testing pending

## Case report

An 86-year-old woman presented with an acute pustular eruption that developed 3 days after initiating an over-the-counter turmeric supplement. She started the supplement for perceived health benefits. Although the labeled serving size recommended three 750 mg capsules daily, the patient reported ingesting 6 capsules per day for 2 consecutive days, totaling 4,500 mg daily. On the third day, she developed a pruritic rash. She discontinued the supplement after 2 days but delayed evaluation for approximately 2 weeks. Her medical history was significant for hypertension treated with valsartan 160 mg daily, which had been stable for 9 years without recent medication changes. She denied any newly initiated prescription medications, antibiotics, or other over-the-counter supplements preceding eruption onset.

**Fig 1:**
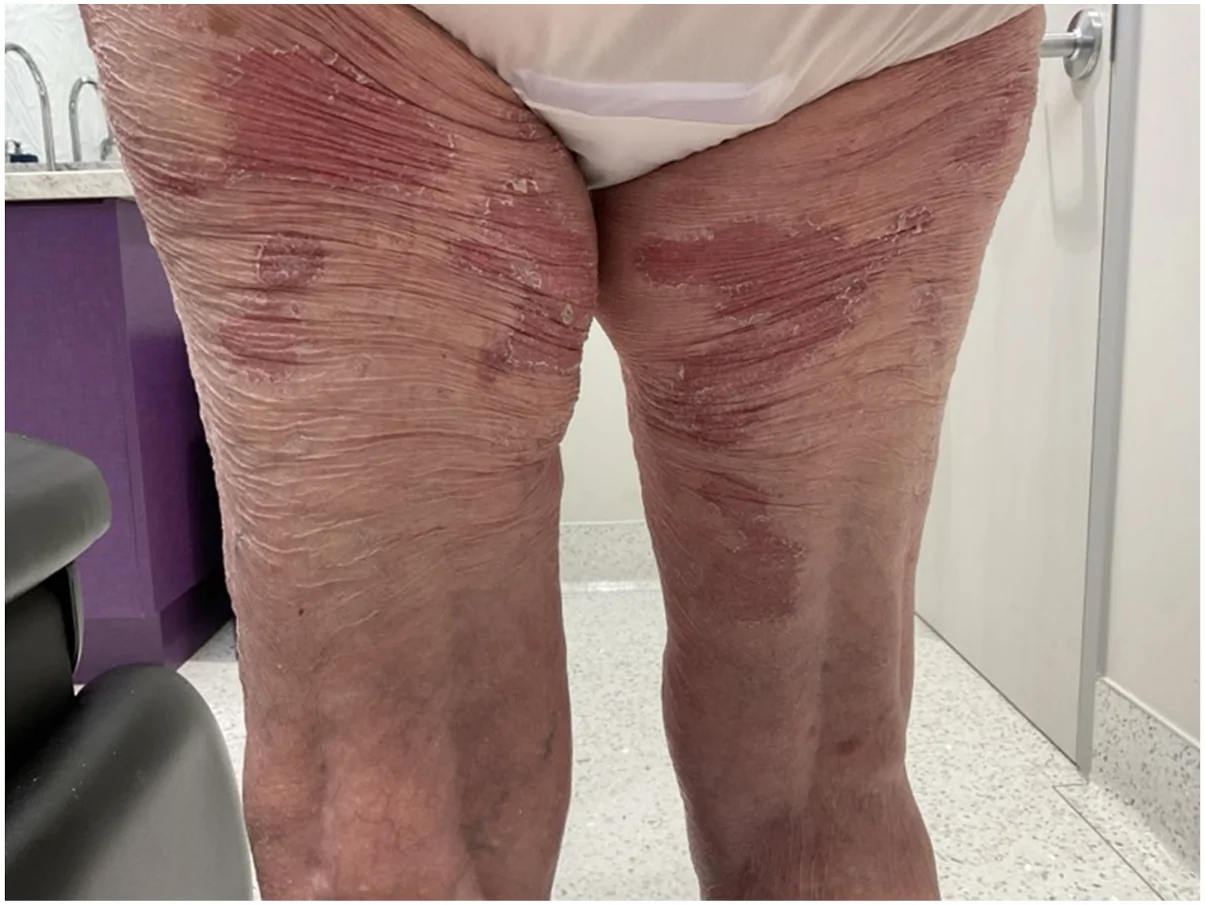
Clinical presentation of turmeric-associated acute generalized exanthematous pustulosis. Residual erythema with scattered monomorphic pustules involving the bilateral posterior thighs.

**Fig 2:**
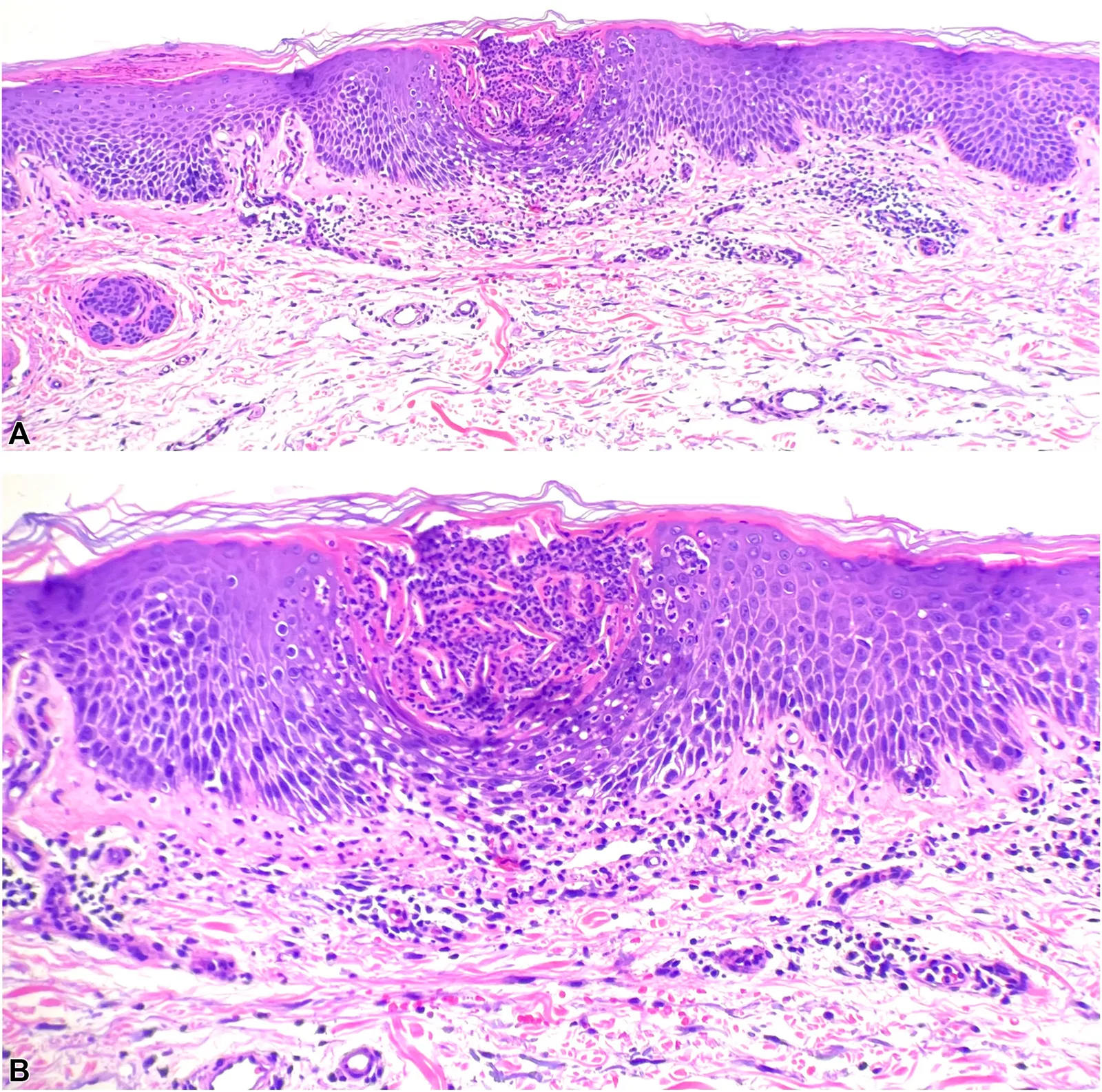
Histopathologic findings. **A,** Subcorneal pustular dermatitis with a subcorneal vesicle containing numerous neutrophils (H&E, × 10). **B,** Higher magnification demonstrating neutrophils within the stratum corneum and a superficial perivascular lymphocytic infiltrate (H&E, × 20).

On examination, residual erythema with scattered resolving monomorphic pustules was observed on the thighs and abdomen (Fig 1). The patient reported feeling feverish at eruption onset. However, no documented fever was present at the time of evaluation approximately 2 weeks later. Complete blood count was within normal limits, with a white blood cell count of 5.3k/mm^3^. A shave biopsy was performed. Histopathologic evaluation demonstrated subcorneal pustular dermatitis with vesicles containing numerous neutrophils (Fig 2). A superficial perivascular lymphocytic infiltrate and small collections of neutrophils admixed with parakeratosis within the stratum corneum were also identified.

Treatment included intramuscular triamcinolone acetonide 40 mg/mL, 1.5 mL, topical clobetasol 0.05% ointment twice daily for 2 weeks, and loratadine 10 mg daily for one month. Clinical improvement was observed following treatment.

Given the rapid onset after short-term high-dose turmeric ingestion, supportive pathology findings, and absence of alternative triggers, the clinical presentation was most consistent with AGEP.

## Discussion

AGEP is characterized by the abrupt eruption of numerous sterile, nonfollicular pustules on an erythematous base. Lesions may be associated with fever and leukocytosis.^9^, ^10^, ^11^ More than 90% of cases are medication-induced, most commonly by antibiotics, with onset typically occurring within 1 to 3 days of exposure.^9^^,^^11^^,^^12^ Most eruptions resolve within 2 weeks after withdrawal of the offending agent.^9^, ^10^, ^11^, ^12^

Histologically, AGEP features spongiform subcorneal and intraepidermal pustules, necrotic keratinocytes, papillary dermal edema, and prominent neutrophilic infiltrate extending into the mid and deep dermis.^13^ Given the clinical and histologic overlap, generalized pustular psoriasis is an important consideration in the differential diagnosis. Generalized pustular psoriasis more commonly demonstrates prominent psoriasiform epidermal hyperplasia and vascular dilation.^14^ The biopsy findings in this patient are consistent with established AGEP features. Application of the EuroSCAR validation criteria yielded a score of 8, consistent with definite AGEP.^9^ Although the patient did not develop fever, leukocytosis, or mucosal involvement, she demonstrated several characteristic clinical findings including diffuse erythema, involvement of trunk and proximal extremities, postpustular desquamation and supportive histopathologic findings including pustular inflammation and epidermal exocytosis. A limitation of this case is that the patient presented approximately 2 weeks after eruption onset, at which time the pustules were already resolving, limiting direct evaluation of the acute phase of the reaction. However, the combination of recent high-dose turmeric ingestion, supportive clinical and pathologic findings, and a EuroSCAR score of 8 remained consistent with AGEP.

Although medications account for most cases of AGEP, nonmedication triggers including dietary supplements have also been reported.^10^^,^^11^ Turmeric and curcumin possess immunomodulatory properties mediated through nuclear factor kappa B signaling and cytokine pathways.^2^ Although generally considered safe, these biologic effects may contribute to immune-mediated cutaneous reactions in susceptible individuals. In this case, eruption onset occurred within 3 days of high-dose turmeric ingestion, at approximately 4500 mg daily, representing the highest reported turmeric exposure among published cases. Although a definitive dose-response relationship has not been established, the rapid onset following short-term high-dose exposure and increasing turmeric doses reported across cases may suggest a dose-dependent contribution to immune activation in susceptible individuals.

Confirmatory patch testing may further strengthen causality assessment in turmeric-associated hypersensitivity reactions. In our case, patch testing was not available at the time of publication, although the patient was referred for further allergy evaluation. Rechallenge was not pursued given concern for recurrence of a severe cutaneous adverse reaction.

Although a reaction to one of the supplement excipients cannot be excluded, turmeric was considered the most likely culprit because it was the only active ingredient in the formulation and has previously been associated with AGEP and other cutaneous hypersensitivity reactions.

This case and prior reports suggest that turmeric may function as a sensitizing agent capable of triggering hypersensitivity reactions. As herbal supplement use increases, clinicians should recognize that over-the-counter products are not inherently benign and may cause severe cutaneous adverse reactions. Given limited regulatory oversight and dosing variability, careful supplement history is essential when evaluating acute pustular eruptions. Turmeric-associated AGEP should be considered in the differential diagnosis of acute pustular eruptions.

## Conflicts of interest

None disclosed.
